# Assessment of anti-malondialdehyde-acetaldehyde antibody frequencies in rheumatoid arthritis with new data from two independent cohorts, meta-analysis, and meta-regression

**DOI:** 10.1186/s13075-023-03180-x

**Published:** 2023-10-05

**Authors:** Lorena Rodriguez-Martinez, Cristina García-Moreno, Eva Perez-Pampin, María J. Gómara, Juan C. Sarmiento-Monroy, Yolanda Lopez-Golán, José A. Gómez-Puerta, Antonio Mera-Varela, Carmen Conde, Raimon Sanmartí, Isabel Haro, Antonio González

**Affiliations:** 1 Experimental and Observational Rheumatology and Rheumatology Unit, Instituto de Investigacion Sanitaria-Hospital Clínico Universitario de Santiago (IDIS), Santiago de Compostela, 15706 Spain; 2 Present Address: Clinical Pharmacology Group, Health Research Institute of Santiago de Compostela (IDIS), Santiago de Compostela, 15706 Spain; 3 Unit of Synthesis and Biomedical Applications of Peptides, Institute of Advanced Chemistry of Catalonia, Consejo Superior de Investigaciones Científicas, (IQAC-CSIC), Barcelona, 08034 Spain; 4 Arthritis Unit, Rheumatology Department, Hospital Clinic of Barcelona, Barcelona, Spain; 5 Department of Medicine, Faculty of Medicine, Universidade de Santiago de Compostela, Santiago de Compostela, 15705 Spain

**Keywords:** Rheumatoid arthritis, Autoantibodies, Biomarker, Post-translational modifications, Heterogeneity, Meta-analysis

## Abstract

**Background:**

Autoantibodies are critical elements in RA pathogenesis and clinical assessment. The anti-malondialdehyde-acetaldehyde (anti-MAA) antibodies are potentially useful because of their claimed high sensitivity for all RA patients, including those lacking RF and anti-CCP antibodies. Therefore, we aimed to replicate these findings.

**Methods:**

We independently attempted replication in Santiago and Barcelona using sera from 517 and 178 RA patients and 272 and 120 healthy controls, respectively. ELISA protocols for anti-MAA antibodies included five antigens (human serum albumin in three formulations, fibrinogen, and a synthetic peptide) and assays for the IgG, IgM, and IgA isotypes. We integrated our results with information found by searching the Web of Science for reports of anti-MAA antibodies in RA. The available patients (4989 in 11 sets) were included in a meta-analysis aimed at heterogeneity between studies. Factors accounting for heterogeneity were assessed with meta-regression.

**Results:**

The sensitivity of anti-MAA antibodies in our RA patients was low, even in seropositive patients, with the percentage of positives below 23% for all ELISA conditions. Our results and bibliographic research showed IgG anti-MAA positive patients ranging from 6 to 92%. The extreme between-studies heterogeneity could be explained (up to 43%) in univariate analysis by sex, African ethnicity, the site of study, or recruitment from the military. The best model, including African ancestry and smoking, explained a high heterogeneity fraction (74%).

**Conclusion:**

Anti-MAA antibody sensitivity is extremely variable between RA patient collections. A substantial fraction of this variability cannot be attributed to ELISA protocols. On the contrary, heterogeneity is determined by complex factors that include African ethnicity, smoking, and sex.

**Supplementary Information:**

The online version contains supplementary material available at 10.1186/s13075-023-03180-x.

## Introduction

Rheumatoid arthritis (RA) is a complex autoimmune disease that causes a substantial burden and presents many unmet needs [1, 2]. One is the scarcity of biomarkers for diagnosis, evaluation, treatment selection, and prognosis. The demand for biomarkers is particularly acute in seronegative patients, lacking the validated RA autoantibodies rheumatoid factor and anti-cyclic citrullinated peptide (anti-CCP) antibodies because these antibodies are valuable for RA diagnosis and prognosis. Therefore, the 2015 discovery of autoantibodies with high sensitivity for this subset of patients was received with keen interest [3, 4], although this claim has been questioned more recently [5–8]. The antibodies in the 2015 study recognized malondialdehyde-acetaldehyde (MAA) protein adducts in more than 80% of seronegative patients and 92% of the whole set of RA patients [4]. Therefore, anti-MAA antibodies were proposed as biomarkers for seronegative patients and a tool for discovering disease mechanisms shared by seronegative and seropositive patients [3].

The search for anti-MAA antibodies in RA was based on the predisposition of RA patients to produce antibodies against post-translational modifications (anti-PTM) [6, 9]. The best-known anti-PTM antibodies are anti-CCP antibodies, which recognize citrullinated proteins (or peptides). Other anti-PTM antibodies in RA patients recognize proteins modified by carbamylation or acetylation. The MAA adducts are suitable candidates for eliciting an antibody response in RA because they are produced by oxidative stress associated with chronic inflammation [10]. Specifically, reactive oxygen species (ROS) in the persistent inflammatory milieu lead to multiple modifications in neighboring molecules and cellular structures [10, 11]. ROS reaction with lipidic membranes triggers lipid peroxidation (LPO). This process results in a series of intermediate compounds that break down into small aldehydes and other ROS. One of the most abundant aldehydes is malondialdehyde (MDA). Another very reactive aldehyde produced in LPO is acetaldehyde (AA). It can also arise from the spontaneous breakdown of MDA or, in smokers, from tobacco smoke [12]. The two aldehydes acting together form MAA adducts when reacting with proteins and other molecules. These adducts are highly immunogenic and differ from adducts separately formed by MDA or AA [13–15].

The possible relationship between MAA adduct abundance and anti-MAA antibodies could explain the described associations of the antibodies with RA features. Specifically, the anti-MAA associations with RA activity, acute phase reactant levels, RA duration, RA-associated interstitial lung disease, and cardiovascular disease could result from increased inflammation and excess oxidative stress [4, 6, 16–18]. However, MAA adduct abundance may also explain the lack of specificity of anti-MAA antibodies for RA. Indeed, anti-MAA-positive patients are observed in alcoholic liver disease [19], atherosclerotic aortic aneurysms [20], cardiovascular disease [21], systemic lupus erythematosus, and osteoarthritis [18]. Nevertheless, anti-MAA antibodies could still be informative as biomarkers for RA. For example, they are much less frequent in spondyloarthritis than in RA, a finding implying they could be used in differential diagnosis [18].

An obstacle to anti-MAA antibody use is the incomplete validation in subsequent RA studies [5, 6, 8, 18, 22, 23]. Some studies replicated the high sensitivity in seropositive and seronegative RA patients [18, 23], but others reported lower frequencies of positive RA patients [5, 6, 8, 22]. Here, we present our anti-MAA results and attempts to increase anti-MAA sensitivity in the first part of the current article. We complemented it by integrating our results with previous studies in the second part. The integration was done with a meta-analysis of the frequency of IgG anti-MAA^+^ patients in all available studies [5, 6, 8, 18, 23]. This meta-analysis was aimed at quantifying and defining heterogeneity. The use of meta-analysis for this purpose is well-established [24–28], but it is less common than summarizing published studies. Therefore, we briefly explain the results. We followed it with meta-regression analyses to identify factors accounting for anti-MAA heterogeneity. We hope the two parts contribute to understanding anti-MAA antibodies in RA patients.

## Material and methods

### Patients and samples

We studied patients with RA according to the 1987 American College of Rheumatology (ACR) [29] or the 2010 ACR/European League Against Rheumatism (EULAR) criteria [30]. The first part of the study was conceived, designed, and carried out independently in two Spanish towns. In Santiago, we analyzed sera from 517 patients with RA and 272 healthy adult controls that have been previously described [31]. The healthy controls (age = 69.7 ± 8.8 years (mean ± SD)) lacked musculoskeletal, inflammatory, or autoimmune diseases, and they were in good general condition, able to self-care, and willing to consent to the study. In Barcelona, we included 178 patients with RA and 120 healthy adult controls (age = 40.5 ± 12.6 years) from the blood bank of the Hospital Clinic of Barcelona, as previously described [32]. Most patients (98.5 and 96.3% in Santiago and Barcelona, respectively) had established RA and the controls were not age and sex matched to the patients.

### Determination of circulating antibodies against MAA by indirect ELISA

Production of MAA proteins and peptides is described in detail in the Supplementary Material. We produced MAA adducts following Thiele et al. [4] protocol with minor modifications. The quality control procedures included assessment of the structures of Hexyl-MAA used as standard (Supplementary Figure 1) and of the chimeric fibrin/filaggrin MAA adducted peptide (Supplementary Figure 2). The antibodies against MAA adducts were determined by indirect ELISA on Nunc Maxisorp plates (Thermo Scientific) coated with 2 μg/well of MAA-modified or unmodified antigen. The coating was done overnight with antigens in 100 μL of 0.1 M bicarbonate buffer pH 9.6 at 4 °C. We used stirring for the incubations and 3 × 200 μL PBS-Tween 20 0.05% (PBS-T) for the washes. The remaining ELISA details varied depending on the experiment.

In Santiago, plate blocking was done with BSA 2% in PBS for 2 h at room temperature (RT) for the main results and casein instead of BSA for alternative results. The patient and control sera at 1:100 dilution in BSA (or casein for the alternative results) 1% + PBS-T were incubated overnight at 4 °C. The secondary antibodies were alkaline phosphatase (AP)-conjugated goat antibodies from Jackson ImmunoResearch. They were directed against human IgG (Heavy + Light chains, or Fcγ-specific for the alternative results), IgM (Fc_5m_-specific), and IgA (α-chain specific). These secondary antibodies were used at 1:5000, 1:8000, and 1:1000 in BSA 1% + PBS-T, respectively. After 1 h at RT, the signal was revealed with p-nitrophenyl phosphate (pNPP) (Pierce, Thermo Scientific) for 30 min in the dark, followed by OD reading at 405 nm in a Multiskan EX Microplate Reader (Thermo Scientific).

In Barcelona, plates were blocked with casein 2% in PBS-T for 1 h at RT. The sera were diluted in casein 0.5% + PBS-T at 1:250 for the IgG and IgA isotypes and 1:500 for the IgM isotype. The diluted sera were incubated for 2 h at RT. The secondary antibodies were horseradish peroxidase (HRP)-conjugated goat antibodies. They were specific to human IgG (Fcγ specific), IgM (Fc_5m_ specific), and IgA (α-chain specific). The three were diluted 1:5000 in casein 2% + PBS-T and incubated for 1 h at RT. The HRP substrate, 3,3′,5,5′-Tetramethylbenzidine (BD Biosciences, USA) was added for 23 min in the dark. The reaction was stopped with 50 μL of 2N H_2_SO_4_ followed by OD reading at 450 nm in a LT4500 microplate reader (Labtech, UK). This protocol was also applied to all antibodies analyzed in Barcelona except the anti-CFF(MAA)P antibodies. They required coating with Neutravidin diluted in PBS (0.5 mg/well, overnight at 4 °C + 1 h at 37 °C) to capture the biotinylated peptides. The two biotinylated peptides, CFF(MAA)P and its native arginine counterpart (CFFP-R), were incubated for 1 h at 1 μg/mL in PBS and 37 °C. The plates were blocked with BSA 2% + PBS-T for 30 min at 37 °C. Next, the sera (at 1:250-fold in RIA buffer supplemented with 10% fetal bovine serum) were incubated for 1 h at 37 °C and then overnight at 4 °C. Afterward, a secondary HRP-conjugated anti-human IgG antibody (diluted 1:4000 in RIA buffer) was developed for 1 h at 37 °C. The substrate was SigmaFast (Sigma-Aldrich, USA), which contains o-phenylenediamine dihydrochloride (OPD). The reaction was stopped with 50 μL of 2N H_2_SO_4_ and read at 492 nm.

In each ELISA, the reactivity to the non-modified antigen was subtracted from the reactivity to the MAA-modified protein/peptide to obtain specific OD values. All samples were assayed in duplicate. Duplicates with less than 10% coefficient of variation (CV) and plates with less than 5% CV in the controls and standards were considered acceptable. In Santiago, antibody levels were expressed in arbitrary units (AU) relative to a 4-parameter logistic regression curve made with serial dilutions of pooled positive sera. In Barcelona, antibody quantification was performed in ng/mL with linear regression by reference to known IgG concentrations (ChromPure Human IgG, whole molecule, Jackson ImmunoResearch). The cutoff for positivity was set at the 98th and 95th percentile of healthy controls in Santiago and Barcelona, respectively.

### Meta-analysis and meta-regression

Supplementary Material contains a description of the general statistical analysis. Here, we detail the random-effects meta-analysis used to understand the heterogeneity of anti-MAA frequencies [24, 25, 27, 28]. The first step was to search for previous reports in the Web of Science (WOS) database (Clarivate Analytics) through the FECYT portal (https://www.recursoscientificos.fecyt.es/). This database includes the Science Citation Index Expanded (SCI-EXPANDED), Conference Proceedings Citation Index – Science (CPCI-S), Book Citation Index – Science (BKCI-S), and the Emerging Sources Citation Index (ESCI). The query terms were “(rheumatoid arthritis OR antibodies) AND malondialdehyde-acetaldehyde.” The search period was from 1990 to 31 October 2022. The first publication was from 1997 and most publications (120/131) were posterior to the study of Thiele et al. [4]. Two authors searched the titles and abstracts of the 131 publications for articles that reported the frequency of anti-MAA^+^ antibodies in RA patients. They selected 28 publications that required an examination of the full text. This process identified six publications with original and non-redundant anti-MAA antibody frequencies in RA patients [4–6, 8, 18, 23]. Analysis of the bibliography of these publications did not reveal any additional sources. One of the publications was a meeting abstract at the time of the search [33], but the full article became available before the final analyses [5]. This article includes four patient sets that join the five previously published studies and our two RA collections. This makes a total of 11 datasets.

The combination of patient sets was done with random effect meta-analysis using the inverse variance for study weights and maximum likelihood for estimation of the between-studies variance (tau-squared, *τ*^2^). This approach is appropriate for heterogeneity-focused meta-analysis and allows likelihood ratio tests for model selection [24, 26, 28]. All the variables reported as proportions (IgG anti-MAA^+^ antibody frequencies, ancestry, sex, smoking, and ACPA positivity) were logit-transformed to stabilize variance [26]. However, the graphs show the corresponding percentages after back transformation. The dispersion of anti-MAA antibody frequencies was assessed with Cochran’s *Q*. Finally, the fraction of the total variance attributed to heterogeneity between studies was estimated with the inconsistency (*I*^2^) statistic.

We included in the meta-regression analyses the available features associated with anti-MAA antibodies in previous reports (*n* = 6) or identified by us as potential confounding factors (*n* = 3). These nine factors were considered in individual and combination models with stepwise meta-regression. The results are reported focusing on the regression coefficient (*β*), its *p*-value (*p*_*β*_), and the three measures of heterogeneity (*Q*, *I*^2^, and *τ*^2^) [24, 25, 27, 28]. These heterogeneity measures were interpreted relative to the model without any factors, particularly in the case of *τ*^2^ [25–28]. This latter relationship is reported as *R*^2^, which is the fraction of between-studies heterogeneity attributed to the model. We used two stepwise meta-regression procedures to identify the best model. The forward selection began with the model without factors and selected the factors for inclusion in increasing order of their *p* values in univariate analyses. The backward elimination procedure started with the complete model. Elimination of factors progressed in decreasing order of their coefficients’ *p*-value in the multivariate model. The fit of the models was assessed at each step with three parameters, log(Likelihood), Akaike information criterion (AIC), and Bayes information criterion (BIC). The best model was identified as significantly better than the previous models and not inferior to the next. The differences were considered significant if *p* < 0.05 in the likelihood ratio test for nested models or ≥ 2 in AIC or BIC. These analyses were done with JASP (https://jasp-stats.org/), which implements the metafor R package [26].

## Results

### Lower anti-HSA-MAA antibody reactivity in RA patients than initially described

**Fig. 1:**
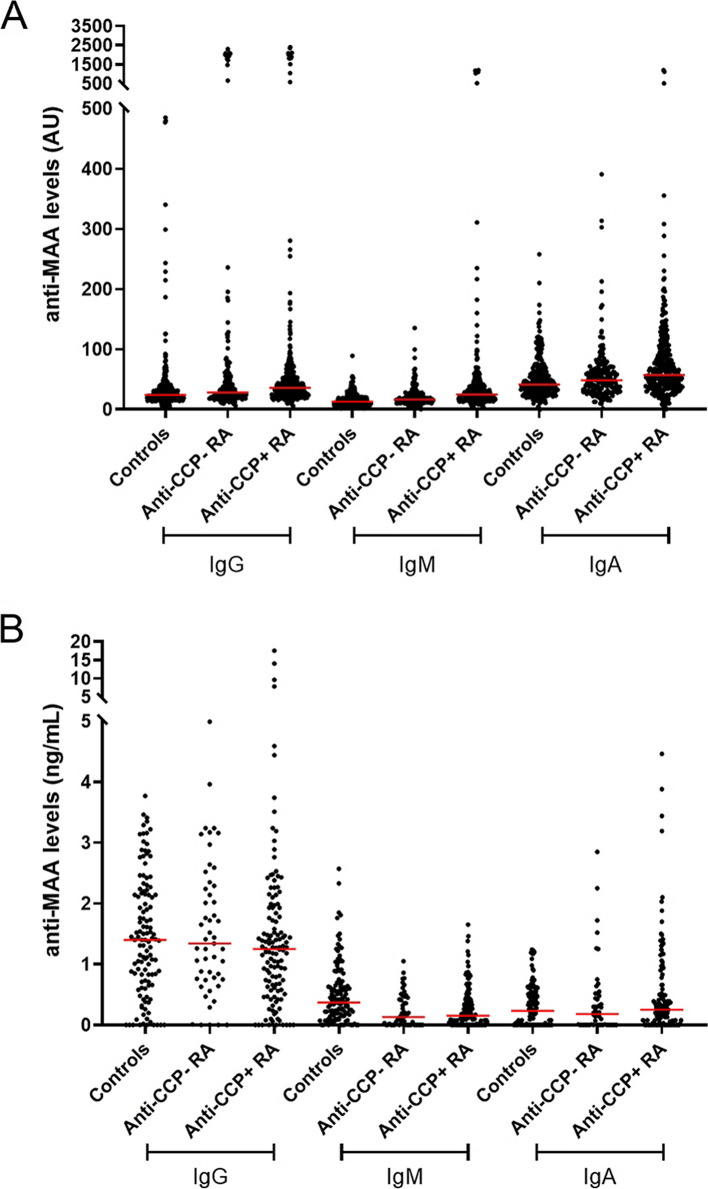
Anti-MAA antibody levels in healthy controls, anti-CCP- RA patients, and anti-CCP + RA patients. The results in **A** and **B** were obtained with complete independence at Santiago and Barcelona, respectively. In **A** 272 healthy controls, 185 anti-CCP^−^ and 332 anti-CCP^+^ RA patients were included. In **B** the numbers were 120, 52, and 127, respectively. Each dot represents a subject; the red horizontal lines represent the median anti-MAA antibody levels

Trying to replicate Thiele et al. results [4], the authors in Santiago determined the anti-HSA-MAA antibodies in sera from 517 RA patients and 272 healthy controls. The results showed higher median levels of anti-MAA antibodies of the three Ig isotypes in the RA patients than in the healthy controls (Fig. 1A and Supplementary Table 1). This fact was reflected in the low frequency of positive patients. Only 6.0%, 14.8%, and 7.2% were positive for IgG, IgM, and IgA anti-MAA antibodies, respectively (Supplementary Table 1). The low positive frequencies were observed in the anti-CCP^+^ and anti-CCP^−^ subgroups (all below 20%). The percentages were considerably lower than in the initial report (Table 1), where the lowest frequency was 38.0% for the IgM anti-MAA antibodies. These results were not attributable to a higher reactivity against the unmodified HSA in RA patients than in controls (Supplementary Figure 3A) or to the threshold for positivity (Supplementary Table 2).

**Table 1:** Contrasts between the current study and the initial report in anti-MAA^+^ patients with RA

Patient set^a^	*N*^b^	IgG anti-MAA^c^ *n* (%)	*p*	IgM anti-MAA^c^ *n* (%)	*p*	IgA anti-MAA^c^ *n* (%)	*p*
All RA							
Santiago	517	31 (6.0)	< 10^−310^	76 (14.8)	< 10^−20^	37 (7.2)	< 10^−150^
Barcelona	178	13 (7.3)	< 10^−180^	1 (0.6)	< 10^−20^	23 (12.9)	< 10^−55^
Thiele, 2015 [4]	1720	1582 (92.0)	Ref	654 (38.0)	Ref	1256 (73.0)	Ref
Anti-CCP^+^							
Santiago	332	18 (5.4)	< 10^−240^	65 (19.7)	< 10^−10^	29 (8.8)	< 10^−110^
Barcelona	127	9 (7.1)	< 10^−150^	1 (0.8)	< 10^−15^	17 (13.4)	< 10^−45^
Thiele, 2015 [4]	1340	1246 (93.0)	Ref	549 (41.0)	Ref	1018 (76.0)	Ref
Anti-CCP^−^							
Santiago	185	13 (7.0)	< 10^−75^	11 (5.9)	< 10^−9^	8 (4.3)	< 10^−35^
Barcelona	51	4 (7.8)	< 10^−35^	0 (0)	< 10^−5^	6 (11.8)	< 10^−10^
Thiele, 2015 [4]	380	334 (87.9)	Ref	110 (28.9)	Ref	239 (62.9)	Ref

^a^Patients with RA from the three collections were considered globally (All RA) and in the anti-CCP^+^ and anti-CCP^−^ subgroups

^b^Abbreviations: *N* = total number of patients in each set; *n* = number of antibody positive patients; % = percentage of antibody positive patients; *p* = *p* value; Ref. = set used as a reference for comparison

^c^The number of anti-MAA antibody positive patients for each immunoglobulin isotype was determined using MAA-modified HSA as antigen and relative to the thresholds obtained with healthy controls in each collection, as detailed in the Material and methods

In an attempt to increase anti-HSA-MAA sensitivity, we applied several modifications that made our methodology more akin to Thiele et al. [4]. Albunorm® 5% (Octapharma, Lachen, Switzerland), a pharmaceutical solution for infusion in which HSA is already solubilized, was employed instead of the lyophilized protein. Protein dialysis for purification and casein buffers for the ELISA were used instead of the AMICON system and BSA buffers, respectively. Two sets of ELISA were done with the modified conditions (Table 2). The first set also included HSA adduction with an increased MDA:AA ratio (4:1). However, the frequency of anti-HSA-MAA^+^ RA patients did not increase in any of the three isotypes (Table 2). The second set was only used for the IgG isotype. It included the modified conditions described above with a 2:1 MDA:AA ratio and an alternative secondary antibody that might reduce background reactivity. However, the fraction of IgG anti-MAA^+^ patients (8.3%) was not different from that of the other protocols (Table 2).

**Table 2:** No difference in anti-HSA-MAA^+^ RA patients between the main ELISA and two modified protocols

Isotype	Anti-MAA^+^ RA patients %^a^			Difference protocols^b^	
	Main	1st	2nd	*p* main *vs.* 1st	*p* main *vs.* 2nd
IgG	14.6	14.9	8.3	0.7	0.4
IgM	6.3	8.3	-	1	-
IgA	6.3	4.2	-	1	-

The main protocol was used in Fig. 1 and Table 1. The 1st modified protocol changed the HSA source, increased the ratio of malondialdehyde to acetaldehyde to 4:1, replaced BSA for casein in the blocking buffer and the adduct purification system. The 2nd modified protocol was as the 1st except for the 2:1 MDA to AA ratio and the anti-Fcγ secondary antibody instead of the anti-(H + L) IgG antibody

^a^Patients and controls, 48 and 48, were randomly selected from the Santiago collection. The cut-off for positivity was specific for each protocol at the estimated 98th percentile

^b^Comparison of the protocols with the McNemar test (with the Yates correction) for paired samples

### Independent assays showed low anti-MAA reactivity in RA patients from Barcelona

Another replication of Thiele et al. was independently performed in Barcelona. The authors assessed anti-MAA antibodies in sera from 178 RA patients and 120 healthy controls recruited locally. The ELISA did not find higher anti-MAA antibody levels in the patients than in the healthy controls (Fig. 1B and Supplementary Table 1). These results were not attributable to increased reactivity against native HSA in RA patients (Supplementary Figure 3B). A fraction of the patients showed IgA antibodies above the highest control levels (Fig. 1B). This was reflected in a significantly increased frequency of IgA anti-MAA^+^ RA patients (12.9%) but not in the other isotypes. These results were independent of the threshold for positivity (Supplementary Table 2) and were not affected by anti-CCP status (Supplementary Table 1). Therefore, the experiments in Barcelona also showed much lower anti-MAA reactivity than initially reported (Table 1).

Additionally, two other MAA-adducted antigens were employed to detect IgG anti-MAA reactivity at Barcelona. The two antigens revealed a significantly higher percentage of antibody-positive RA patients than HSA-MAA (Table 3). The anti-MAA antibodies detected with Fib-MAA or CFF(MAA)P were positive in about 20% of RA patients versus 7.3% with HSA-MAA. However, the frequency of positive RA patients did not approach the initially described, not even considering the reactivity against any of the three MAA adducts, 34.3% (Supplementary Fig. 4). The low positivity was not attributable to a higher reactivity against the native antigens in the RA patients than in the controls (Supplementary Figure 3B-D).

**Table 3:** Higher frequency of positive RA patients for IgG anti-alternative MAA-modified antigens than anti-HSA-MAA

Patient set^a^	*N*^b^	anti-Fib-MAA *n* (%)	*p*^c^	anti-CFF(MAA)P *n* (%)	*p*^c^	anti-HSA-MAA *n* (%)
All RA	178	32 (18.0)	0.002	34 (19.1)	0.001	13 (7.3)
Anti-CCP^+^	127	26 (20.5)	0.002	28 (22.0)	0.0008	9 (7.1)
Anti-CCP^−^	51	6 (11.8)	0.7	6 (11.8)	0.7	4 (7.8)

^a^Patients with RA from the Barcelona collection were considered globally (All RA) and in the anti-CCP^+^ and anti-CCP^−^ subgroups

^b^Abbreviations are as in Table 1 and *Fib-MAA*, MAA-modified fibrinogen; *CFF(MAA)P*, chimeric fibrin/filaggrin synthetic peptide containing MAA; *HSA-MAA*, MAA-modified human serum albumin

^c^*P* values of the comparison with the anti-HSA-MAA percentage. Table 1 already presents anti-HSA-MAA results, but they are shown here for easy reference

### Demographic and clinical features associated with anti-HSA-MAA status

The low frequency of anti-HSA-MAA^+^ in our patients limited the power of association analyses. Despite this, we replicated several associations in the previously reported directions (Table 4). Specifically, RA patients in Santiago showed an association of the IgG anti-MAA antibodies with male sex [4, 18]; the IgM anti-MAA with ever-smokers, anti-CCP, and RF antibodies [4, 8]; and the IgA antibodies with disease duration and RF antibodies [4, 8].

**Table 4:** Features associated with anti-HSA-MAA^+^ antibody status in RA patients from Santiago and Barcelona

	IgG-anti-MAA		IgM-anti-MAA		IgA-MAA	
	OR (95% CI)^a^	*p*	OR (95% CI)	*p*	OR (95% CI)	*p*
Santiago						
Sex (men)	3.3 (1.3–8.6)	0.014	1.1 (0.7–1.9)	0.6	0.9 (0.5–1.7)	0.8
Age at diagnosis	1.0 (1.0–1.0)	0.057	1.0 (1.0–1.0)	0.5	1.0 (1.0–1.0)	0.2
Disease evolution	1.0 (1.0–1.1)	0.085	1.0 (1.0–1.0)	0.4	1.0 (1.0–1.1)	7.9 × 10^−3^
Smoking (ever)	0.8 (0.2–3.0)	0.7	2.8 (1.4–5.7)	4.1 × 10^−3^	1.7 (0.6–4.5)	0.3
Anti-CCP^+^	0.7 (0.3–1.4)	0.3	3.9 (2.0–7.6)	9.0 × 10^−5^	2.0 (0.9–4.5)	0.095
RF^+^	0.7 (0.3–1.5)	0.3	3.5 (1.8–6.6)	1.4 × 10^−4^	2.6 (1.1–6.2)	0.03
SE	1.0 (0.5–2.0)	0.9	1.2 (0.7–2.0)	0.5	0.8 (0.4–1.6)	0.6
*PTPN22*	1.1 (0.5–2.5)	0.8	0.7 (0.4–1.4)	0.3	0.8 (0.3–1.7)	0.5
Barcelona						
Sex (men)	1.7 (0.6–5.4)	0.3	-^b^	-	0.7 (0.3–1.6)	0.4
Age at diagnosis	1.0 (0.9–1.0)	0.063	0.9 (0.8–1.1)	0.5	1.0 (1.0–1.0)	0.9
Disease evolution	0.9 (0.8–1.1)	0.4	0.8 (0.3–1.7)	0.5	1.1 (1.0–1.1)	0.2
Smoking (ever)	0.8 (0.2–3.0)	0.7	-	-	0.4 (0.1–1.1)	0.067
Anti-CCP^+^	0.9 (0.3–3.3)	0.9	-	-	1.1 (0.4–3.0)	0.9
RF^+^	1.0 (0.3–3.5)	0.9	-	-	0.9 (0.4–2.3)	0.9

Results of the logistic regression analysis. Sex, age at diagnosis, and disease evolution results are from univariate models. The other features were analyzed with sex, age, and disease duration as covariates

^a^*Abbreviations*: *OR* odds ratio, *CI* confidence interval, *RF* rheumatoid factor, *SE* alleles of the shared epitope in the *HLA-DRB1* gene, *PTPN22* risk allele of the *rs2476601* SNP in the *PTPN22* gene

^b^The number of antibody positive patients was too low for analysis

### Meta-analysis of IgG anti-MAA antibody prevalence in RA patients

The low anti-MAA positivity found with multiple assays in our two laboratories led us to hypothesize that other factors beyond the ELISA protocol may be responsible. Therefore, we systematically searched the bibliography for other potential causes of anti-MAA variability. Using the World of Science (WOS) database, we found six articles containing non-redundant information on the percentage of anti-MAA^+^ RA patients (Table 5). The six publications reported results for the IgG isotype, whereas the other isotypes were analyzed only in three articles [4, 8, 18]. Therefore, only the IgG isotype was considered for meta-analysis. Five publications included a patient collection; the remaining publication contained four patient sets, each from a different continent [5]. Therefore, we considered nine patient sets from previous studies and the two collections reported here. These 11 sets included 67 to 1720 RA patients each, totaling 4989 patients in all.

**Table 5:** Characteristics of the studies reporting IgG anti-MAA antibody frequency in RA patients

Characteristics	Thiele, 2015 [4]^a^	Mikuls, 2018 [18]	Mikuls, 2020 [8]	Petro, 2021 [23]	Grönwall, 2021 [6]	de Moel, 2023 [5] NLD/FNS/JPN/SA	Santiago	Barcelona
Number of patients	1720	284	214	1229	403	103/100/174/67	517	178
IgG anti-MAA^+^, %	92	80	22	67	44	29/29/22/53^b^	6	7
European, %	78	75	58	89	100	100/0/0/0	100	100
African, %	16	17	Yes^c^	6	0	0/0/0/100	0	0
Age, mean (SD)	63 (11)	59 (12)	37 (8)^d^	57 (13)	50 (13)	58/48/60/49^e^	61 (14)	59 (13)
Women, %	9	37	48	79	70	66/79/82/89	77	79
Ever smokers, %	80	62^f^	33	52	72	60/84/28/12	20	46
Military, predominantly	Yes	Yes	Yes	No	No	No	No	No
anti-CCP^+^, %	78	85^f^	71	55	69	100	64	71
IgG anti-MAA^+^/anti-CCP^+^, %	93	83	22^b^	74	49	29/29/22/53^b^	5	7
Percentile IgG anti-MAA^g^	99	95	90	(67)^g^	85	97.5^ g^	98	95
Years with RA, mean (SD)	12 (12)	13 (10)^f^	7 (4)	-	1^ h^	1.3/7.7/7.5/0^e^	14 (11)	6 (5)

^a^Studies are referred to by the first author and publication year. de Moel (2023) [5] reports the frequency of IgG anti-MAA antibodies for four sets of patients: *NLD*, Dutch from the Netherlands; *FNS*, First Nations People from Canada; *JPN*, Japanese; *SA*, South Africans

^b^In these patient sets, we considered IgG anti-MAA^+^ % = IgG anti-MAA^+^/anti-CCP^+^ % for meta-regression because de Moel et al. (2023) [5] study included only anti-CCP^+^ RA patients and Mikuls et al. (2020) [8] did not report IgG anti-MAA^+^/anti-CCP^+^ %. We performed sensitivity analyses to check this procedure produced consistent results

^c^An unspecified fraction of the 42% non-EU patients have African ancestry. In meta-regression, we tested 32, 24, 16, and 8% with similar results (Supplementary Table 4) and reported the data for 8%

^d^Age at diagnosis. The age used for meta-regression was 44 years resulting from adding the mean number of years with RA

^e^Median values in years

^f^Data obtained from (PMID: 24,782,175; https://doi.org/10.1002/art.38348)

^g^Percentiles of anti-MAA antibodies used to define the threshold for positive anti-MAA antibody

^h^All patients in this collection have early RA (< 1 year)

Remarkably, the percentage of IgG anti-MAA^+^ patients showed considerable variability ranging from 6 to 92% (Table 5).

**Fig. 2:**
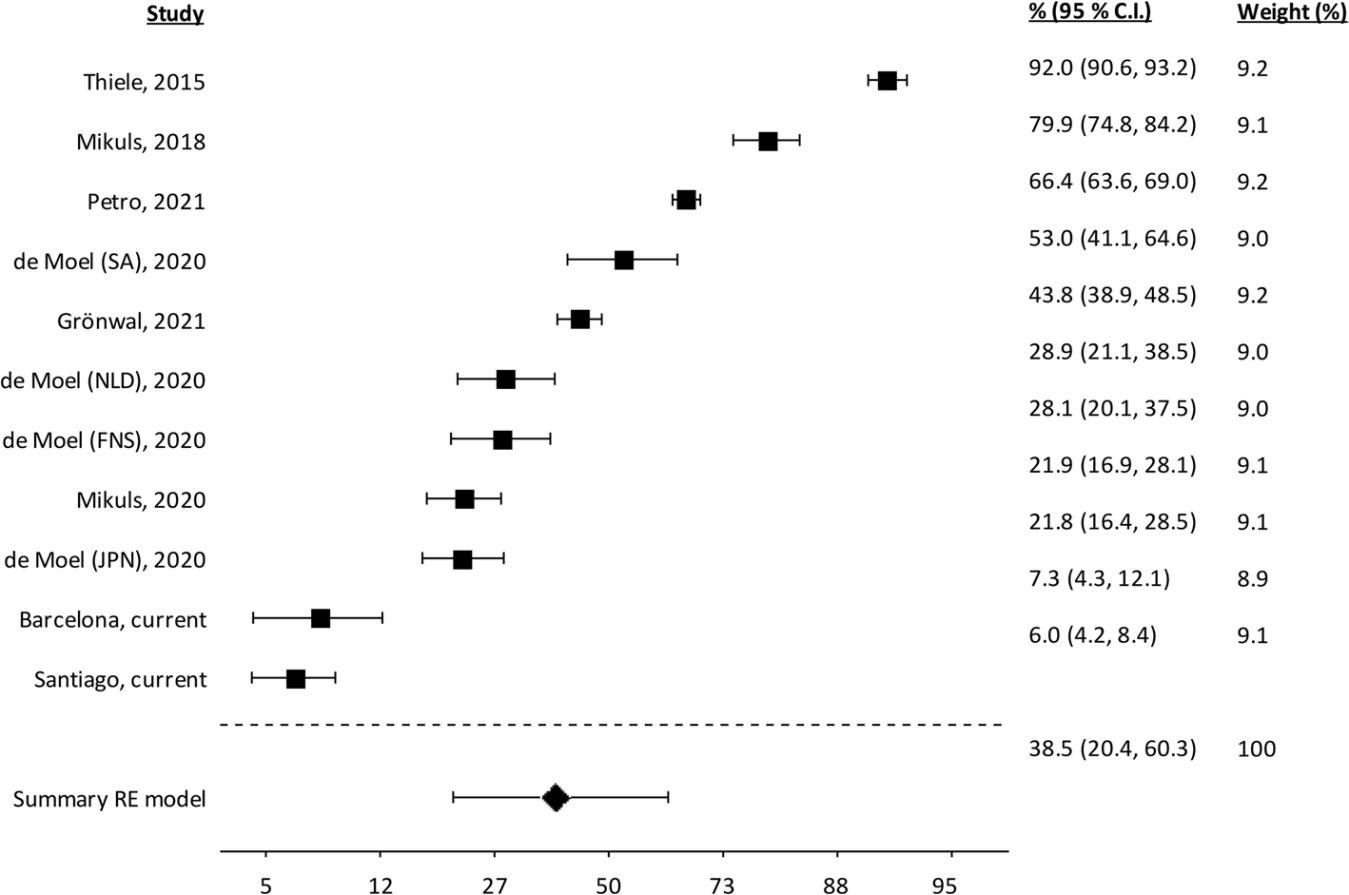
Random effect meta-analysis of the frequency of IgG anti-MAA^+^ in RA patients. The patient sets are identified by the first author and year of publication. The four RA patient sets in de Moel (2023) [5] are FNS, First Nations People from Canada; SA, South Africans; NLD, Dutch from the Netherlands; and JPN, Japanese. The size of the squares is proportional to the weight of each patient collection (provided in the last column). The *X*-axis is on the logit scale. RE, random effects. 95% CI, 95% confidence interval

The large variability required a random effects meta-analysis focused on quantifying heterogeneity. The forest plot showed a wide dispersion of frequencies characterized by confidence intervals with minor overlap (Fig. 2). The uniform distribution indicated the absence of outlier studies (formally excluded in addition) that could disproportionately influence the results. The extreme variability was reflected in a very significant heterogeneity test (Cochran *Q p* = 1.9 × 10^−270^) and high *I*^2^ value (99.2%, and Supplementary Table 3).

As a result of considering the site of analysis, a critical insight was obtained. The Leiden laboratory found frequencies ranging from 22 to 53% in four patient sets [5]. Similarly, the four studies at the University of Nebraska reported IgG anti-MAA^+^ frequencies extending from 22 to 92% [4, 8, 18, 23]. These examples of wide variability within the same laboratory reinforced our hypothesis of significant determinants of anti-MAA positivity in RA patients beyond ELISA specificities.

### Meta-regression reveals significant factors contributing to between-studies heterogeneity

We conducted meta-regression analyses to investigate factors that could be associated with heterogeneity [24–28]. We were mindful of the potential for bias in this type of analysis. Therefore, we exclusively analyzed factors with evidence of association in previous studies and with available data from 10 or 11 patient sets. There were six factors with these characteristics: African ethnicity, anti-CCP positivity, smoking, male sex, RA disease duration, and current age (Table 5). In addition, we considered three peculiarities of the studies as potential confounders: the percentile defining anti-MAA positivity, predominant recruitment among the military, and sharing the analysis site (Table 5). The nine factors were individually analyzed by random effects meta-regression. In the second part, they were included in the search for the best multivariate model using forward selection and backward elimination stepwise regression analyses (Table 6).

**Table 6:** Meta-regression of factors for IgG anti-MAA^+^ frequency in RA patients

RE meta-regression model	*β*	*p*_*β*_	*τ*^2^ (95% CI)	*R*^2^
Without factor	-	-	2.23 (1.18, 7.61)	-
Previously associated factors				
AF ancestry (%)^a^	0.25	0.032	1.56 (0.89, 6.43)	0.300
Anti-CCP (%)	− 0.06	0.8	2.21 (1.26, 9.09)	0.009
Women (%)	− 0.81	0.0070	1.32 (0.75, 5.53)	0.408
Smokers (%)	0.61	0.10	1.77 (1.01, 7.36)	0.206
Age (years)	0.03	0.6	2.18 (1.25, 8.99)	0.022
Time since diagnosis (y)	0.04	0.7	2.27 (1.28, 10.52)	0.000
Potential confounders				
Percentile	− 0.03	0.5	2.15 (1.23, 8.81)	0.036
Military (yes)	1.83	0.032	1.56 (0.89, 6.44)	0.300
Site (N/O)^b^	2.03	0.0044	1.27 (0.72, 5.27)	0.431
Site (L/O’)^c^	− 0.43	0.6	2.18 (1.25, 8.99)	0.022
Best forward selection model^d^				
Site (N/O)^b^	2.03	0.0044	1.27 (0.72, 5.27)	0.430
Best backward elimination model^d^				
AF ancestry (%)	0.35	4.1 × 10^−6^	0.58 (0.35, 3.12)	0.740
Smokers (%)	0.94	2.7 × 10^−5^		

A significant association is reflected in a significant *β* coefficient, a decrease in the residual between-studies heterogeneity (*τ*^2^) relative to the model without factors, and a large fraction of the between-studies heterogeneity explained (*R*^2^)

*Abbreviations*: *RE* random effects, *τ*^*2*^ squared tau, between study heterogeneity, *95% CI* 95% confidence interval, *AF* African, *Anti-CCP* anti-cyclic citrullinated peptide antibodies

^a^Four percentages of African ancestry (32, 24, 16, and 8%) were considered for the Mikuls (2020) [8] study (Supplementary Table 4). Here, we show the results corresponding to 8%

^b^N = Nebraska = Thiele, 2015 [4] + Mikuls, 2018 [18] + Mikuls, 2020 [8] + Petro 2021 [23]; O = all the other patient sets

^c^L = Leiden = de Moel, 2023 [5]; O’ = all the other patient sets

^d^Best models obtained with forward and backward stepwise procedures. The *β* coefficients (and *p*_*β*_) correspond to the indicated factor. The other parameters correspond to the model

Two features were associated with anti-MAA^+^ patients in univariate meta-regression models. The strongest association was observed with sex. The men’s frequency was associated with more anti-MAA^+^ RA patients (*β* = 0.81, *p* = 0.0070), and this factor accounted for 40.8% of the between-studies residual heterogeneity (Table 6; other measures of heterogeneity are in Supplementary Table 3). Sex showed a broad range of frequencies, with women representing 9–89% of RA patients (Table 5). The other factor was the fraction of patients with African ancestry (Table 6). It was significantly associated with increased anti-MAA^+^ frequency (*β* = 0.25, *p* = 0.032) and accounted for 30.0% of between-study heterogeneity. Information on African ancestry was retrievable from ten patient sets ranging from 0 to 100% (Table 5). As for the remaining study, Mikuls et al. [8], did not specify the African subgroup within the 42% of non-European RA patients. Therefore, we tested four equally spaced values that yielded similar results (Supplementary Table 4). Among the other factors, the percentage of ever-smokers showed a trend towards an increased frequency of anti-MAA^+^ patients (*β* = 0.61, *p* = 0.10, Table 6). Sex, African ethnicity, and smoking were all associated in the same direction as previously reported.

Two peculiarities of the studies were also associated with anti-MAA^+^ patients (Table 6). The most associated characteristic was the N/O classification of the analysis sites (*β* = 2.03, *p* = 0.0044). It was followed by predominant patient recruitment from the US military (*p* = 0.032, Table 6). This circumstance is a particularity of three University of Nebraska studies. The third peculiarity, the threshold for antibody positivity, was not significant (not shown).

The two stepwise meta-regression selections produced different best models. The forward selection led to a model with only the N/O factor, which was included in the first step. The second step would have added sex, but the model did not improve fit. Moreover, the coefficients indicated that site N/O and sex were redundant (Supplementary Table 5). In contrast, the backward elimination procedure identified a model with a reinforced association of African ancestry and smoking relative to the corresponding 1-factor analyses (4.1 × 10^−6^ for AF ancestry and 2.7 × 10^−5^ for smoking) accounting for a substantial fraction of between-studies heterogeneity (*R*^2^ = 0.740). Indeed, the best backward model showed a significantly improved fit relative to the best forward selection model (the AIC and BIC values were 6.2 and 5.8 units lower than for the best forward model).

## Discussion

Our work highlights the extreme variability in anti-MAA^+^ RA patients and the complex network of factors behind it. The lowest frequencies of anti-MAA^+^ RA patients were found in our two laboratories, each with its own anti-MAA ELISA, patients, and controls. The findings were consistent across immunoglobulin isotypes, antigen preparations, and ELISA modifications. Therefore, we hypothesized that other factors beyond the technique should explain the heterogeneity. We gathered support for this hypothesis from two laboratories reporting highly variable anti-MAA frequencies in separate patient sets [4, 5, 8, 18, 23]. Our investigation of heterogeneity revealed a complex causality network, including factors previously reported in individual studies [4, 8, 18] and interaction between them. Although the best model explained 74% of between-studies heterogeneity, the remaining heterogeneity was still highly significant, suggesting that more factors could still participate.

First, we considered the ELISA technical aspects because anti-MAA antibodies can only be determined with assays developed in each laboratory. For this reason, we initially suspected the low frequency of anti-MAA^+^ patients was due to suboptimal ELISA conditions. However, we did not observe any evidence to support this interpretation. We checked the quality of MAA adducts and tried multiple ELISA modifications without any significant sensitivity increase. Notably, the tested conditions included five distinct substrates for MAA adducts. The trials were performed in our two laboratories without knowledge of each other, although the two groups sought and obtained advice from the Nebraska laboratory. However, none of the suggested modifications approached the initially reported frequencies of anti-MAA antibodies [4, 18].

A systematic bibliographic investigation revealed other studies that joined to our results covered the complete range of IgG anti-MAA^+^ frequencies [4–6, 8, 18, 23] without any identifiable confluence point. This distribution already suggested a complex causality behind the observed heterogeneity. The bibliographic investigation also disclosed variable antibody frequencies in patient sets analyzed in the same laboratory. The frequencies were 22 to 92% at the University of Nebraska [4, 8, 18, 23] and 22 to 53% at the Leiden Medical Center [5]. These two examples of within-laboratory variability reinforced the hypothesis that other factors beyond the ELISA account for the main fraction of the heterogeneity. In addition, the clustering of patient sets at these two analysis sites could represent a potential source of confounding. We identified two other potential confounders: the predominant inclusion of military [4, 5, 8, 18] and the choice of variable percentile thresholds to define antibody positivity.

The meta-analysis findings motivated a meta-regression analysis to search for causal factors [24–28]. However, a frequently commented pitfall of meta-regression is false associations due to ecological bias [27, 34], which occurs when the analyzed and causal factors coincide in the same studies. The likelihood of this artifact increases with the number of features analyzed, the small number of studies, and the performance of subanalyses. Therefore, we prevented ecological bias by considering, without subanalyses, only the six characteristics previously associated with anti-MAA antibodies and available from ≥ 10 patient collections.

The association of African ethnicity with IgG anti-MAA antibodies is the most clearly defined. The association has been reported in three previous studies [4, 5, 18]. In these studies, African ancestry patients showed high levels and positivity of IgG anti-MAA antibodies. In two of them, the same associations were observed for IgA anti-MAA antibodies [4, 18]. However, the contribution of African ancestry to between-study heterogeneity was not evident before our analysis because the frequency of IgG anti-MAA^+^ patients did not correlate with the fraction of African subjects [4, 5, 8, 18, 23]. Our meta-regression illuminated this point. First, the results showed that African ancestry was associated with between-study heterogeneity. In addition, the best meta-regression model showed evidence of an interaction between African ethnicity and smoking, which explained the absence of a linear correlation.

Two possible mechanisms behind African ethnicity association with IgG anti-MAA antibodies were suggested by de Moel et al. [5]. One is a propensity to produce more Ig and RA autoantibodies. This mechanism is demonstrated by the higher levels and frequency of other anti-PTM autoantibodies, besides anti-MAA antibodies, in black South African patients and healthy controls [5]. This finding was partly explained by higher serum IgG levels in South Africans than in other groups [5]. In other studies, African ethnicity was associated with more RA autoantibodies [35] and higher serum Ig levels than other ethnic groups [36]. The second mechanism is the high frequency of RA patients without treatment in the South African group [5]. A similar circumstance could contribute to high anti-MAA antibodies in the other African ancestry studies because disparities in treatment, access to care, and socioeconomic status are common in African-American RA patients [37, 38].

Regarding smoking, this feature was associated with IgM and IgA anti-MAA antibodies in previous studies [4, 18] and with the positivity of IgM anti-MAA antibodies in our Santiago RA patients. However, no individual study has observed an association with anti-MAA antibodies of the IgG isotype. This fact calls for prudence in interpreting the results. Nevertheless, the direction of association makes sense from a pathogenic perspective. Indeed, the abundance of aldehydes in cigarette smoke [12] and the induction of autoimmunity and autoantibodies by smoking [1, 2, 39, 40] are consistent with an increased frequency of anti-MAA^+^ patients. In more detail, smoking is the most established environmental RA risk factor [1, 2]. It acts in the early phases of pathogenesis, well before symptoms develop, and is linked to the induction of autoimmunity [41, 42]. These effects of smoking are reflected in the simultaneous presence of multiple autoantibodies [43–45].

Association of sex with anti-MAA antibodies has been reported in two previous studies [4, 18] and replicated in our Santiago patients. In all three cases, men presented higher levels than women [4, 18]. The meta-regression analysis was concordant with these associations. However, the “sex” factor was indistinguishable from “predominance of the military” and “analysis site at Nebraska University” in multivariate analyses. We can explain this finding because men were the minor fraction (< 35%) of RA patients [5, 6, 23] except in the three studies that included many military personnel (> 51%), done at the University of Nebraska [4, 8, 18]. Therefore, part of the association of anti-MAA^+^ frequency with two potential confounders may be explained by the prominent presence of men in some RA patient collections.

This example shows that discordant results in meta-regression and individual studies are possible. The two types of analysis have unique strengths and limitations [24, 27, 34]. The contrast is manifest for sex, as commented, and for anti-CCP antibodies, which were associated with anti-MAA antibodies in four of the seven patient collections with anti-CCP^+^ and anti-CCP^−^ patients [4, 6, 8], but not in the meta-regression.

It is necessary to highlight the considerable between-studies heterogeneity that was not accounted for as a limitation of our study. This limitation is reflected in the significant *Q* = 210, *P*_*Q*_ = 5.7 × 10^−41^, and the remarkable inconsistency *I*^2^ = 98.5% remaining in the best backward meta-regression model. As such, we still need to find other factors explaining heterogeneity. Some might be features identified in previous studies but not included in meta-regression because they were absent in other collections, such as disease activity and specific treatments. Furthermore, the path to the definition of the contribution of each factor will require more patient sets. Ideally, future studies should include all relevant features and anti-MAA antibodies of both IgG, IgA, and IgM isotypes.

## Conclusions

Our work has shown that technical details are not the critical cause of variability in anti-MAA^+^ RA patients. However, technical factors will only be definitively excluded if a standardized assay becomes available. Independently of the ELISA, our analyses indicate that irreproducibility will be challenging to control. Indeed, the extraordinary heterogeneity of anti-MAA^+^ frequencies and the evidence of complex determinants invite skepticism about the interpretability of anti-MAA antibodies.

In conclusion, we have exposed the wide variability in anti-MAA^+^ RA patient frequencies and its dependency on a complex network of factors beyond the technical ones. The factors include African ancestry, smoking, and the patient’s sex, which were identifiable by meta-regression or in replicated individual studies.

### Supplementary Information

**Additional file 1: Supplementary methods. Supplementary Table 1.** Comparison of anti-HSA-MAA antibody levels and frequencies between the controls and the RA patients and between the anti-CCP- and anti-CCP+ patients in the Santiago and Barcelona collections. **Supplementary Table 2.** Percentage of positive RA patients corresponding to different cut-off values in the anti-HSA-MAA ELISA performed in Santiago and Barcelona. **Supplementary Table 3.** Additional heterogeneity parameters from Table 5 meta-regression models. **Supplementary Table 4.** Key meta-regression models considering four AF ancestry percentages for Mikuls et al. [8]. **Supplementary Table 5.** Fit of the nested multivariate meta-regression models. **Supplementary Table 6.** Similar to Table 6 in the main text but with the IgG anti-MAA+/anti-CCP+ % in RA patients. **Supplementary Figure 1.** Characterization by 1H NMR of 1-hexyl-4-methyl-1,4-dihydro-3,5-pyridine-dicarboxaldehyde (Hexyl-MAA). **Supplementary Figure 2.** Characterization of the chimeric fibrin/filaggrin MAA adducted peptide. **Supplementary Figure 3.** Comparison of the IgG reactivity against native and MAA-modified antigens between healthy controls (○, empty circles) and RA patients (●, filled circles). **Supplementary Figure 4.** Relative frequency of IgG anti-MAA+ RA patients with the three MAA adducts assayed in Barcelona.

## Data Availability

The datasets used and analyzed during the current study are available from the corresponding authors on reasonable request.

## Abbreviations

^1^H NMR: Proton nuclear magnetic resonance
AA: Acetaldehyde
ACPA: Anti-citrullinated protein/peptide antibodies
ACR: American College of Rheumatology
AIC: Akaike information criterion
Anti-CCP: Anti-cyclic citrullinated peptides antibodies
Anti-MAA: Anti-malondialdehyde-acetaldehyde antibodies
Anti-PTM: Antibodies against post-translational modifications
AU: Arbitrary units
BIC: Bayes information criterion
BKCI-S: Book Citation Index – Science
BSA: Bovine serum albumin
CDCl_3_: Deuterated chloroform
CFF(MAA)P: Chimeric fibrin/filaggrin synthetic peptide containing malondialdehyde-acetaldehyde
CFFP-R: Unmodified chimeric fibrin/filaggrin peptide
CI: Confidence interval
CPCI-S: Conference Proceedings Citation Index – Science
CV: Coefficient of variation
DTPA: Diethylenetriaminepentaacetic acid
ELISA: Enzyme-linked immunosorbent assay
ESCI: Emerging Sources Citation Index
ES-MS: Electrospray Ionization Mass Spectrometry
EU: European
EULAR: European League Against Rheumatism
Fib-MAA: Malondialdehyde-acetaldehyde modified fibrinogen
FNS: First Nations People from Canada
HC: Healthy controls
Hexyl-MAA: 1-Hexyl-4-methyl-1,4-dihydro-3,5-pyridine-dicarboxaldehyde
HPLC: High performance liquid chromatography
HSA: Human serum albumin
HSA-MAA: Malondialdehyde-acetaldehyde modified human serum albumin
Ig: Immunoglobulin
JPN: Japanese
Log(L): Log(Likelihood)
LPO: Lipid peroxidation
MAA: Malondialdehyde-acetaldehyde
MDA: Malondialdehyde
NLD: Dutch from the Netherlands
OD: Optical density
OR: Odds ratio
PA: Phytic acid
PEG: Polyethylene glycol
*PTPN22*: Risk allele of the rs2476601 SNP in the *protein tyrosine phosphatase non-receptor type 22* gene
RA: Rheumatoid arthritis
RE: Random effects
RF: Rheumatoid factor
ROS: Reactive oxygen species
SA: South Africans
SCI-EXPANDED: Science Citation Index Expanded
SE: Alleles of the shared epitope in the *HLA-DRB1* gene
TMS: Tetramethylsilane
WOS: Web of Science
