# Supplementary material for: Assessment of anti-malondialdehyde-acetaldehyde antibody frequencies in rheumatoid arthritis with new data from two independent cohorts, meta-analysis, and meta-regression
Source: Arthritis Res Ther. 2023 Oct 5;25:192. doi: 10.1186/s13075-023-03180-x (PMC10552211; doi:10.1186/s13075-023-03180-x)

# Assessment of anti-malondialdehyde-acetaldehyde antibody frequencies in rheumatoid arthritis with new data from two independent cohorts, meta-analysis, and meta-regression

Lorena Rodriguez-Martinez ^1#^, Cristina García-Moreno ^2#^, Eva Perez-Pampin ^1^, María J. Gómara ^2^, Juan C. Sarmiento-Monroy ^3^, Yolanda Lopez-Golán ^1^, José A. Gómez-Puerta ^3^, Antonio Mera-Varela ^1.4^, Carmen Conde ^1^, Raimon Sanmartí ^3^, Isabel Haro ^2*^, Antonio González ^1*^

^1^ Experimental and Observational Rheumatology and Rheumatology Unit, Instituto de Investigacion Sanitaria-Hospital Clínico Universitario de Santiago (IDIS), 15706 Santiago de Compostela, Spain

^2^ Unit of Synthesis and Biomedical Applications of Peptides, Institute of Advanced Chemistry of Catalonia, Consejo Superior de Investigaciones Científicas, (IQAC-CSIC), 08034 Barcelona, Spain.­

^3^ Arthritis Unit, Rheumatology Department, Hospital Clinic of Barcelona, Spain.

^4^ Department of Medicine, Faculty of Medicine, Universidade de Santiago de Compostela, 15705 Santiago de Compostela, Spain

^#^ These two authors contributed equally

^*^ Correspondence: agmartinezp@ser.es; Tel.: +34-981950903 or [isabel.haro@iqac.csic.es](mailto:isabel.haro@iqac.csic.es)

## SUPPLEMENTARY MATERIAL

## SUPPLEMENTARY METHODS

### Chemical synthesis of MDA and hexyl-MAA

Malondialdehyde was obtained as sodium salt according to Kikugawa *et al.* [[1](#_ENREF_1)] in the two laboratories. Briefly, 5 mL of 1,1,3,3-tetra-methoxy-propane (Sigma-Merck) was stirred with 2.5 mL of HCl 1 M at room temperature for 30 min. The solution was then cooled on ice for 15 min and adjusted to pH 10 with NaOH 6 M. The sodium salt (MDA·Na) was precipitated by adding 100 mL of cold acetone and allowing it to cool for 1 h. The crystalline MDA·Na was collected by filtration, frozen at -20 ºC for 1.5 h, and purified by recrystallization with cold water-acetone (1:1). The resulting MDA·Na was dried overnight in a non-vacuum desiccator cabinet and stored at -20ºC protected from light.

The synthetic analog of MAA-lysine 1-hexyl-4-methyl-1,4-dihydro-3,5-pyridine-dicarboxaldehyde (Hexyl-MAA) was synthesized and purified following the procedure described by Xu *et al.* (Xu, 1997) and characterized by proton nuclear magnetic resonance spectroscopy (^1^H NMR). The ^1^H NMR spectra were recorded in deuterated chloroform (CDCl_3_) on a Varian Mercury 300 spectrometer (Varian Inc.) in Santiago or a Bruker Ascend 400 MHz NMR spectrometer (Bruker) in Barcelona, respectively. The chemical shifts (δ) were expressed in ppm relative to tetramethylsilane (TMS; δ = 0.0 ppm) and were used to confirm the compound structure (Supplementary Figure 1). We used the validated Hexyl-MAA as a reference to calculate the adducted proteins ratio [[2](#_ENREF_2)].

### MAA-adducted proteins/peptides

We produced five different antigens containing MAA adducts following Thiele *et al.* [[3](#_ENREF_3)] protocol with minor modifications. First, 2 mg/mL of the protein, 2 mM freshly prepared MDA, 1 mM AA, 2 mM diethylenetriamine pentaacetic acid (DTPA), and 2 mM phytic acid (PA) (all from Sigma)  were combined in  0.1 M phosphate buffer pH 7.2 and kept to react for 72 h at 37 ºC in the dark. The unmodified antigen controls were prepared in the same way but without MDA and AA. At the end of the incubation, proteins were extensively dialyzed with 0.1 M phosphate buffer pH 7.2 at 4ºC for 24 h and concentrated. All preparations were filtered through a 0.22 μM syringe filter to avoid protein aggregates. We determined protein concentration with Bradford’s method and verified the MAA adducts with the fluorescence emission spectrum. All MAA adducts showed a ratio > 1 relative to the Hexyl-MAA reference at 470 nm. In Santiago, two preparations of human serum albumin (HSA) were MAA-adducted: Lyophilized HSA (Sigma-Merck, A8763) for the main results; and Albunorm® 5% (Octapharma, Lachen, Switzerland), a pharmaceutical solution, for the alternative results. Also, two protocols were used for dialysis: the 30 kDa Amicon Pro Purification System (Merck) for the main results and the Pur-A-Lyzer Dyalisis (Sigma) kit for alternative results. The other three MAA adducts (two proteins and a peptide) were prepared in Barcelona. The proteins were lyophilized HSA (Sigma-Merck, A1887) and fibrinogen from human plasma (Sigma-Merck 341576). They were used to produce HSA-MAA and Fib-MAA, respectively. The third antigen was a chimeric fibrin/filaggrin peptide modified with MAA at lysine amino acids (CFF(MAA)P) and biotinylated at its terminal amino group with a biotin derivative containing two polyethylene glycol (PEG) chains. This derivatization strategy allows better antigenic exposure of the peptide in ELISA plates. The peptide backbone was selected from previous work [[4](#_ENREF_4), [5](#_ENREF_5)]. It was obtained following the Solid Phase Peptide Synthesis procedure [[4](#_ENREF_4)]. Its primary structure as well as its analytical characterization by HPLC and mass spectrometry are shown in Supplementary Figure 2. The protocol of adduction in Barcelona included dialysis in 3.5K MWCO Slide-A-Lyzer G3 Dialysis (Thermo Scientific, Ref A52966) cassettes.

### General statistical analysis

The differences between anti-MAA levels were assessed with the Mann-Whitney test, whereas anti-MAA^+^ frequencies were compared between groups with the chi-squared test and between ELISA protocols in the same subjects with the McNemar test. In addition, we explored the association of serological, genetic, and clinical features with anti-MAA antibody positivity using multivariate logistic regression. Odds ratios (ORs) and 95% confidence intervals (CIs) adjusted for sex, age at diagnosis, and time since diagnosis are reported except for these three features that were analyzed without confounders. Statistical analysis was conducted with Statistica v7.0 (Statsoft, Tulsa, OK). Graphical representation of data was performed with Statistica, GraphPad Prism v8.0.1 (La Jolla, California), and eulerAPE v3 (https://doi.org/10.1371/journal.pone.0101717). P-values of 0.05 or less were considered statistically significant.

## References

1. Kikugawa K, Ido Y. Studies on peroxidized lipids. V. Formation and characterization of 1,4-dihydropyridine-3,5-dicarbaldehydes as model of fluorescent components in lipofuscin. Lipids. 1984;19(8):600-8.

2. Thiele GM, Klassen LW, Tuma DJ. Formation and immunological properties of aldehyde-derived protein adducts following alcohol consumption. Methods Mol Biol. 2008;447:235-57.

3. Thiele GM, Duryee MJ, Anderson DR, Klassen LW, Mohring SM, Young KA et al. Malondialdehyde-acetaldehyde adducts and anti-malondialdehyde-acetaldehyde antibodies in rheumatoid arthritis. Arthritis Rheumatol. 2015;67(3):645-55.

4. Pérez ML, Gómara MJ, Ercilla G, Sanmartí R, Haro I. Antibodies to citrullinated human fibrinogen synthetic peptides in diagnosing rheumatoid arthritis. J Med Chem. 2007;50(15):3573-84.

5. Sanmarti R, Graell E, Perez ML, Ercilla G, Vinas O, Gomez-Puerta JA et al. Diagnostic and prognostic value of antibodies against chimeric fibrin/filaggrin citrullinated synthetic peptides in rheumatoid arthritis. Arthritis Res Ther. 2009;11(5):R135.

**Supplementary Table 1.** Comparison of anti-HSA-MAA antibody levels and frequencies between the controls and the RA patients and between the anti-CCP^-^ and anti-CCP^+^ patients in the Santiago and Barcelona collections ^a^.

|  | IgG anti-MAA | | IgM anti-MAA | | IgA anti-MAA | |
| --- | --- | --- | --- | --- | --- | --- |
| Group | median (IQ) | +/N (%) | median (IQ) | +/N (%) | median (IQ) | +/N (%) |
| Santiago |  |  |  |  |  |  |
| HC | 23.9 (17.2-34.4) | 6/272 (2.2) | 12.9 (8.2-19.5) | 6/272 (2.2) | 41.3 (28.9-62.1) | 6/272 (2.2) |
| RA patients | 32.8 (21.6-52.8) | 31/517 (6.0) | 21.1 (14.1-34.0) | 76/515 (14.8) | 53.5 (35.0-79.4) | 37/512 (7.2) |
| *p* HC *vs.* RA ^b^ | 1.5 x10^-11^ | 0.017 | 8.4 x10^-26^ | 4.2 x10^-8^ | 1.7 x10^-6^ | 0.0033 |
| RA anti-CCP^-^ | 27.8 (19.2-51.5) | 13/185 (7.0) | 16.4 (11.5-25.3) | 11/185 (5.9) | 48.2 (30.9-66.9) | 8/184 (4.3) |
| RA anti-CCP^+^ | 35.8 (23.8-52.8) | 18/332 (5.4) | 24.6 (16.5-38.3) | 65/330 (19.7) | 57.1 (37.1-89.6) | 29/328 (8.8) |
| *p* + *vs.* - ^b^ | 0.0019 | 0.5 | 1.9 x10^-12^ | 2.4 x10^-5^ | 0.00027 | 0.06 |
| Barcelona |  |  |  |  |  |  |
| HC | 1.40 (0.71-2.16) | 6/120 (5.0) | 0.37 (0.16-0.68) | 6/120 (5.0) | 0.23 (0-0.53) | 6/120 (5.0) |
| RA patients | 1.26 (0.66-1.99) | 13/178 (7.3) | 0.15 (0-0.42) | 1/178 (0.56) | 0.21 (0-0.49) | 23/178 (12.9) |
| *p* HC *vs.* RA ^b^ | 0.5 | 0.5 | 2.3 x10^-7^ | 0.018 | 0.6 | 0.028 |
| RA anti-CCP^-^ | 1.34 (0.75-2.29) | 4/51 (7.8) | 0.13 (0-0.45) | 0/51 (0) | 0.18 (0-0.48) | 6/51 (11.8) |
| RA anti-CCP^+^ | 1.25 (0.61-1.93) | 9/127 (7.1) | 0.15 (0-0.41) | 1/127 (0.8) | 0.25 (0.03-0.50) | 17/127 (13.4) |
| *p* + *vs.* - ^b^ | 0.3 | 1.0 | 0.5 | 1.0 | 0.2 | 1.0 |

^a^ The levels and frequency of positives are not directly comparable between the two collections because of the differences in the ELISA and the thresholds for positivity. These thresholds are the 98^th^ and 95^th^ percentile of the HC in Santiago and Barcelona, respectively.

^b^ The comparisons were done with the Mann-Whitney tests for the antibody levels and the chi squared test for the 2x2 contingency table.

**Supplementary Table 2.** Percentage of positive RA patients corresponding to different cut-off values in the anti-HSA-MAA ELISA performed in Santiago and Barcelona.

|  | IgG | | IgM | | IgA | |
| --- | --- | --- | --- | --- | --- | --- |
| Cut-offs ^a^ | Santiago | Barcelona | Santiago | Barcelona | Santiago | Barcelona |
| Percentile |  |  |  |  |  |  |
| 95^th^ | 11.0 | 7.3 | 23.9 | 0.6 | 12.3 | 12.9 |
| 90^th^ | 21.7 | 11.2 | 30.5 | 1.7 | 16.0 | 19.1 |
| 85^th^ | 31.1 | 11.8 | 41.7 | 3.4 | 23.6 | 21.3 |
| Max. Y-J ^b^ |  |  |  |  |  |  |
| 100 |  |  |  |  |  | 11.8 |
| 80.5 | 42.9 |  |  |  |  |  |
| 73.3 |  |  |  | 55.6 |  |  |
| 72.1 |  |  | 62.7 |  |  |  |
| 64.0 |  |  |  |  | 55.5 |  |
| 45.8 |  | 63.5 |  |  |  |  |

^a^ Two sets of thresholds were evaluated, predefined percentiles and the percentiles that maximized the Youden-J statistic. The latter were specific for each ELISA. All percentiles were defined in the corresponding healthy control sets.

^b^ Max. Y-J = percentiles that maximize the Youden-J statistic, which is the point of best balance between sensitivity and specificity when these two parameters are given the same weight.

**Supplementary Table 3:** Additional heterogeneity parameters from Table 5 meta-regression models ^a^.

| RE meta-regression model ^b^ | Q | p_Q_ | I² (95 % CI) |
| --- | --- | --- | --- |
| without factor | 1288 | 1.9x10 ^-270^ | 99.2 (98.5, 99.8) |
| Previously associated factors |  |  |  |
| AF ancestry (%) | 795 | 2.7x10 ^-165^ | 98.9 (98.0, 99.7) |
| Anti-CCP (%) | 1202 | 4.6x10 ^-253^ | 99.2 (98.6, 99.8) |
| Women (%) | 731 | 1.5x10 ^-154^ | 98.4 (97.2, 99.6) |
| Ever smokers (%) | 785 | 2.8x10 ^-163^ | 99.0 (98.2, 99.8) |
| Age (y) | 943 | 3.9x10 ^-197^ | 99.2 (98.5, 99.8) |
| Time since diagnosis (y) | 1029 | 9.1x10 ^-217^ | 98.8 (97.8, 99.7) |
| Potential confounders |  |  |  |
| Percentile | 1286 | 3.9x10^-271^ | 98.9 (98.1, 99.7) |
| Military (yes) | 987 | 1.5x10^-189^ | 98.7 (97.8, 99.7) |
| Site (N/O) | 679 | 2.1x10^-140^ | 98.5 (97.3, 99.6) |
| Site (L/O') | 1127 | 8.4x10^-237^ | 99.2 (98.6, 99.8) |
| Best forward selection model | 679 | 2.1x10^-140^ | 98.5 (97.3, 99.6) |
| Best backward elimination model | 210 | 5.7x10^-41^ | 98.5 (97.3, 99.6) |

^a^ A significant contribution of a factor is evidenced in a decrease in both, the overall heterogeneity (Q) relative to the model without factors and the between-studies component of heterogeneity (I²).

^b^ The RE meta-regression models are shown in the same order as Table 5.

Abbreviations: RE = random effects; Q = Crochan's Q and its p value, which are measures of the overall heterogeneity; I² = inconsistency, which is interpreted as the fraction of between-studies heterogeneity in the overall heterogeneity; 95 % CI = 95 % confidence interval; AF = African; Anti-CCP = anti-cyclic citrullinated peptides antibodies; N = Nebraska = Thiele, 2015 + Mikuls, 2018 + Mikuls, 2020 + Petro 2021; O = all the other patient sets except N; L = Leiden = de Moel, 2023; O' = all the other patient sets except L.

**Supplementary Table 4.** Key meta-regression models considering four AF ancestry percentages for Mikuls *et al.* 2020 ^a^.

| RE meta-regression model | β_AF_ | p_βAF_ | β_Sm_ | p_βSm_ |
| --- | --- | --- | --- | --- |
| AF ancestry 1-factor models |  |  |  |  |
| Mikuls 2020 = 8 % | 0.25 | 0.032 | - | - |
| Mikuls 2020 = 16 % | 0.24 | 0.040 | - | - |
| Mikuls 2020 = 24 % | 0.23 | 0.046 | - | - |
| Mikuls 2020 = 32 % | 0.23 | 0.052 | - | - |
| Backward elimination models |  |  |  |  |
| Mikuls 2020 = 8 % | 0.35 | 4.1 x10^-6^ | 0.94 | 2.7 x10^-5^ |
| Mikuls 2020 = 16 % | 0.35 | 1.0 x10^-5^ | 0.95 | 4.2 x10^-5^ |
| Mikuls 2020 = 24 % | 0.34 | 1.9 x10^-5^ | 0.96 | 6.0 x10^-5^ |
| Mikuls 2020 = 32 % | 0.33 | 3.3 x10^-5^ | 0.96 | 8.2 x10^-5^ |

^a^ β_AF_ = coefficient of AF ancestry (%), and p_βAF_ = its p value; β_Sm_ = coefficient of Smokers (%), and p_βSm_ = its p value. All other conventions and abbreviations are as in Table 5.

**Supplementary Table 5.** Fit of the nested multivariate meta-regression models ^a^.

| stepwise RE meta-regression models | Log(L) | p_LRT_ | AIC | BIC |
| --- | --- | --- | --- | --- |
| Forward selection |  |  |  |  |
| without factors | -20.1 |  | 44.2 | 45.0 |
| + Site (N/O) | -17.1 | 0.014 | 40.1 | 41.3 |
| + Women | -16.4 | 0.2 | 40.8 | 42.4 |
| Backward elimination |  |  |  |  |
| all factors | -8.4 | 0.7 | 34.8 | 38.4 |
| - anti-CCP | -8.5 | 0.3 | 33.1 | 36.2 |
| - Age | -9.1 | 0.06 | 32.1 | 34.9 |
| - Site (N/O) | -10.9 | 0.3 | 33.9 | 36.3 |
| - Military | -11.5 | 0.08 | 33.0 | 35.0 |
| - Women | -13.0 | 0.0013 | 33.9 | 35.5 |
| - Smokers | -18.2 |  | 42.3 | 43.5 |

^a^ The best fit is defined as the simplest model in a series of nested models that shows a significantly lower loglikelihood (tested with the likelihood ratio test). Alternative definitions are obtained with the lowest AIC or BIC, where a 2 units lower value is considered a better fit. The three definitions were concordant. Note that comparisons in the forward selection procedure are done with the model above, whereas in backward elimination, they are done with the model below.

Abbreviations: RE = random effects; Log(L) = log(Likelihood); pLTR = p of the likelihood ratio test; AIC = Akaike Information Criterion; BIC = Bayesian Information Criterion.

**Supplementary Table 6.** Similar to Table 6 in the main text but with the IgG anti-MAA^+^/anti-CCP^+^ % in RA patients ^a^

| RE meta-regression model | β | p_β_ | τ² (95 % CI) | R² |
| --- | --- | --- | --- | --- |
| without factor | - | - | 2.51 (1.33, 8.61) | - |
| Previously associated factors |  |  |  |  |
| AF ancestry (%) | 0.26 | 0.042 | 1.81 (1.03, 7.50) | 0.279 |
| Anti-CCP (%) | -0.09 | 0.7 | 2.47 (1.41, 10.24) | 0.016 |
| Women (%) | -0.83 | 0.0098 | 1.54 (0.88, 6.47) | 0.387 |
| Smokers (%) | 0.66 | 0.088 | 1.97 (1.12, 8.22) | 0.215 |
| Age (y) | 0.04 | 0.7 | 2.46 (1.41, 10.19) | 0.020 |
| Time since diagnosis (y) | 0.04 | 0.7 | 2.50 (1.40, 11.62) | 0.004 |
| Potential confounders |  |  |  |  |
| Percentile ^c^ | -0.04 | 0.4 | 2.36 (1.35, 9.74) | 0.025 |
| Military (yes) | 1.89 | 0.039 | 1.80 (1.02, 7.46) | 0.283 |
| Site (N/O) ^d^ | 2.20 | 0.0032 | 1.38 (0.78, 5,80) | 0.450 |
| Site (L/O') ^e^ | -0.54 | 0.6 | 2.44 (1.39, 10.10) | 0.028 |
| Best forward selection model ^f^ |  |  |  |  |
| Site (N/O) ^d^ | 2.20 | 0.0032 | 1.38 (0.78, 5,80) | 0.450 |
| Best backward elimination model ^f^ |  |  |  |  |
| AF ancestry (%) | 0.37 | 1.2 x10^-5^ | 0.69 (0.42, 3.70) | 0.729 |
| Smokers (%) | 1.01 | 4.1 x10^-5^ |  |  |

^a^ All conventions and abbreviations are as in Table 5. The 11 patient sets were included. Mikuls *et al.* 2020 study was given 22 % as the IgG anti-MAA+/anti-CCP+ fraction.

**Supplementary Figure 1:** **Characterization by 1H NMR of 1-hexyl-4-methyl-1,4-dihydro-3,5-pyridine-dicarboxaldehyde (Hexyl-MAA).**

The synthesis and characterization of hexyl-MAA were independently performed in Santiago **(A)** and Barcelona **(B)**. The spectrum in **A** was recorded with a Varian Mercury 300 spectrometer. The signals in decreasing order of δ value are: 9.2 (s), 6.6 (s), 3.9 (c), 3.4 (t), 1.2-1.8 (m), 1.1 (d), 0.9 (t) and correspond to peaks A-F. The peak without a letter corresponds to the CDCl_3_ signal. Lowercase letters indicate the multiplicity of peaks (s, singlet; d, doublet; t, triplet, q, quartet and m, multiplet). The spectrum in **B** was recorded in a Bruker Ascend 400 MHz NMR spectrometer in CDCl_3_ and the δ values are indicated in the figure.

**A**


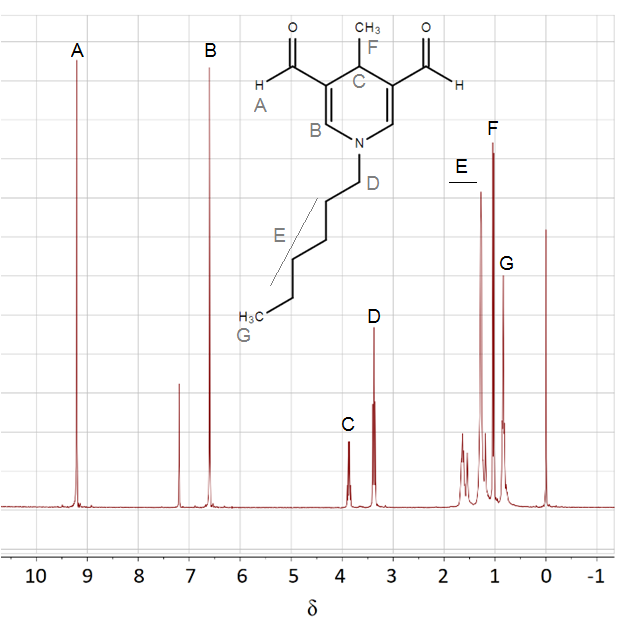


**B**

**
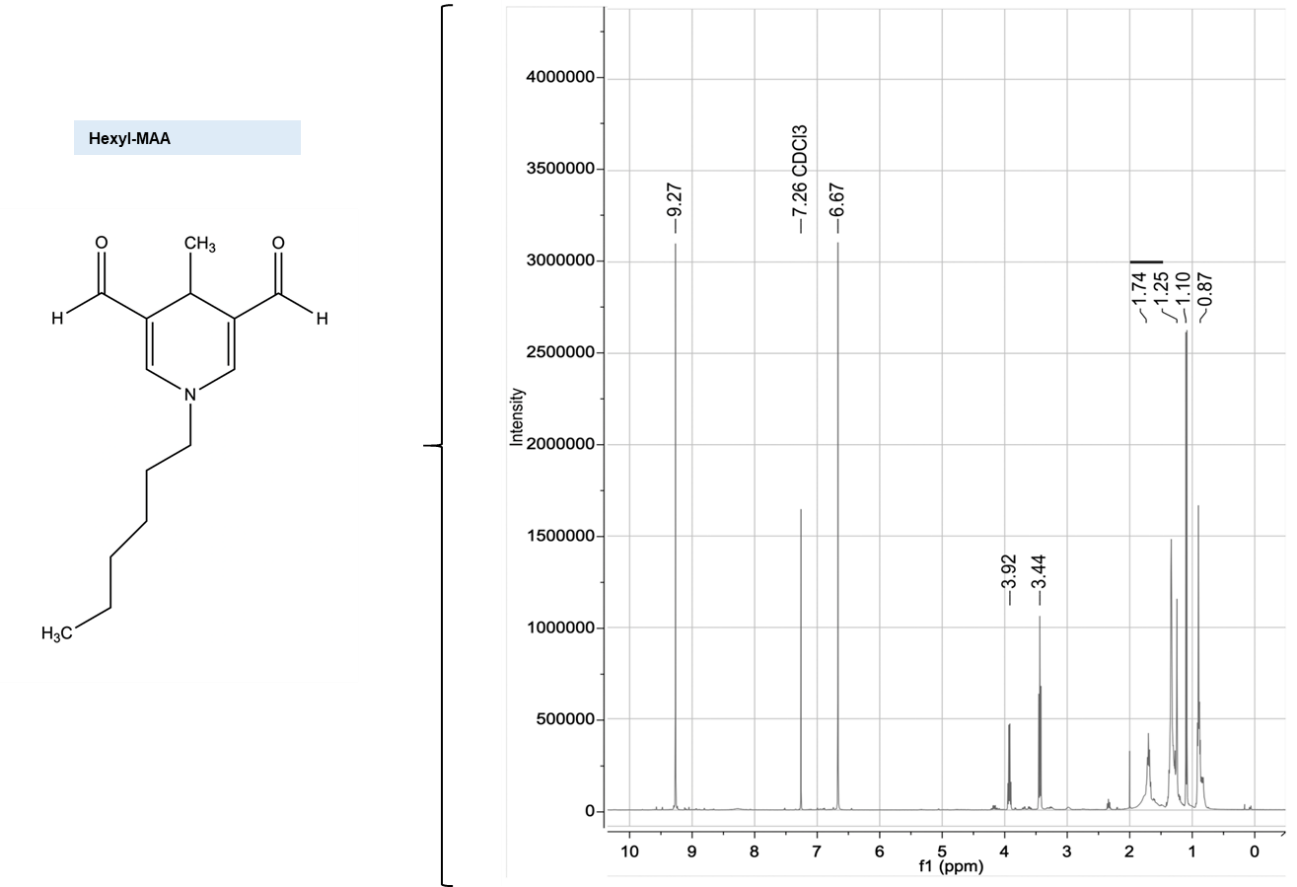
**

**Supplementary Figure 2: Characterization of the chimeric fibrin/filaggrin MAA adducted peptide.**

Typical HPLC chromatogram A) and ES-MS spectra B) of the CFF(MAA)P peptide are shown.

**
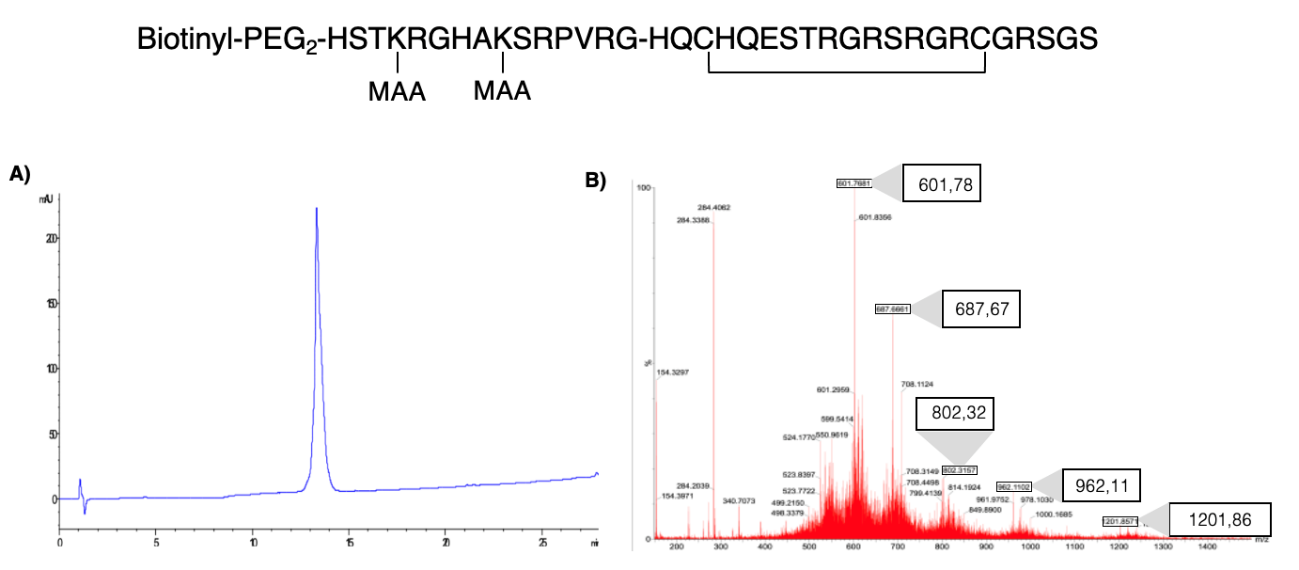
**

Notes:

A) The peptide was eluted on a C18 column under the following HPLC conditions: Eluent A = 0.05% TFA in water; eluent B = 0.05% TFA in acetonitrile. Linear gradient 5-95% of B into A over 20 min at a flow rate of 1 mL/min.

B) Theoretical m/z of the CFF(MAA)P peptide are: [M+4H]^+4^=1202,83, [M+5H]^+5^=962,47; [M+6H]^+6^=802,23; [M+7H]^+7^=687,76; [M+8H]^+8^=601,92. The experimental m/z registered were: [M+4H]^+4^=1201,86, [M+5H]^+5^=962,11; [M+6H]^+6^=802,32; [M+7H]^+7^=687,67; [M+8H]^+8^=601,77.

**Supplementary Figure 3: Comparison of the IgG reactivity against native and MAA-modified antigens between healthy controls (○, empty circles) and RA patients (●, filled circles).** The panels show the optical density (OD) measurements in the ELISA against native HSA and HSA-MAA obtained in Santiago A) and Barcelona B), and against native Fib and Fib-MAA C) and against the CFFP-R and CFF(MAA)P peptides D). Red lines and error bars correspond to median and interquartile range values, respectively.


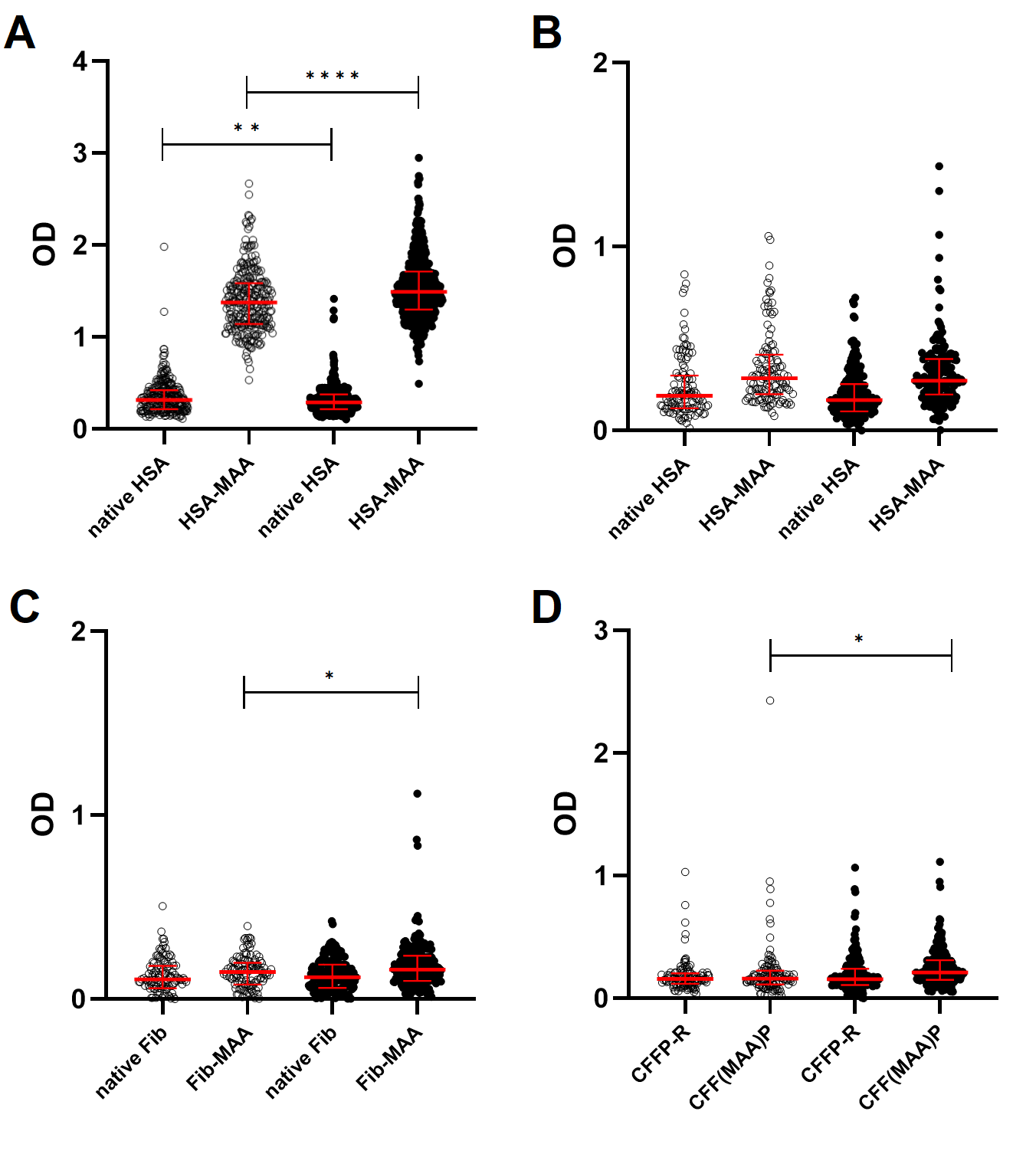


The Mann-Whitney test showed significantly lower IgG reactivity against native HSA in RA patients than HC from Santiago (A). None of the other reactivities against native antigens showed significant differences. Therefore, a higher reactivity against native antigens in the RA patients did not account for the low frequencies of anti-MAA antibodies detected in Santiago or Barcelona. On the contrary, three comparisons showed a higher reactivity against MAA modified antigens in RA patients than in healthy controls (A, C and D).

**Supplementary Figure 4:** **Relative frequency of IgG anti-MAA^+^ RA patients with the three MAA adducts assayed in Barcelona.** The Venn diagram represents the percentage of positive patients for each assay. Among the positives for any of them, 65.7 % of the 178 RA patients from the Barcelona cohort were negative in the three assays. This is indicated in the legend.


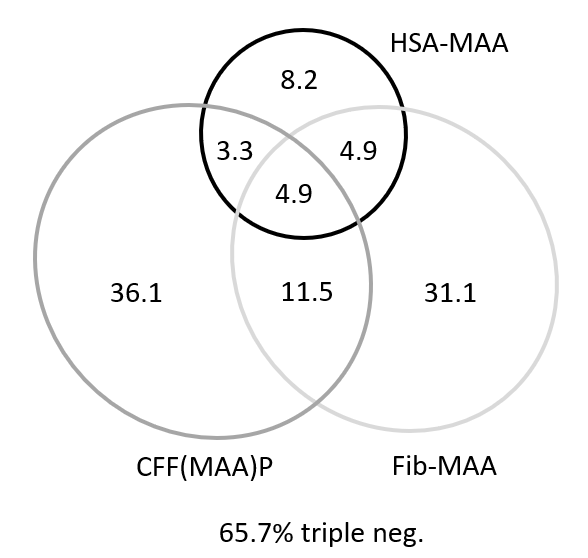

Supplement: Supplementary file 1 — Additional file 1: Supplementary methods. Supplementary Table 1. Comparison of anti-HSA-MAA antibody levels and frequencies between the controls and the RA patients and between the anti-CCP- and anti-CCP+ patients in the Santiago and Barcelona collections. Supplementary Table 2. Percentage of positive RA patients corresponding to different cut-off values in the anti-HSA-MAA ELISA performed in Santiago and Barcelona. Supplementary Table 3. Additional heterogeneity parameters from Table 5 meta-regression models. Supplementary Table 4. Key meta-regression models considering four AF ancestry percentages for Mikuls et al. [8]. Supplementary Table 5. Fit of the nested multivariate meta-regression models. Supplementary Table 6. Similar to Table 6 in the main text but with the IgG anti-MAA+/anti-CCP+ % in RA patients. Supplementary Figure 1. Characterization by 1H NMR of 1-hexyl-4-methyl-1,4-dihydro-3,5-pyridine-dicarboxaldehyde (Hexyl-MAA). Supplementary Figure 2. Characterization of the chimeric fibrin/filaggrin MAA adducted peptide. Supplementary Figure 3. Comparison of the IgG reactivity against native and MAA-modified antigens between healthy controls (○, empty circles) and RA patients (●, filled circles). Supplementary Figure 4. Relative frequency of IgG anti-MAA+ RA patients with the three MAA adducts assayed in Barcelona. [file 13075_2023_3180_MOESM1_ESM.docx]
